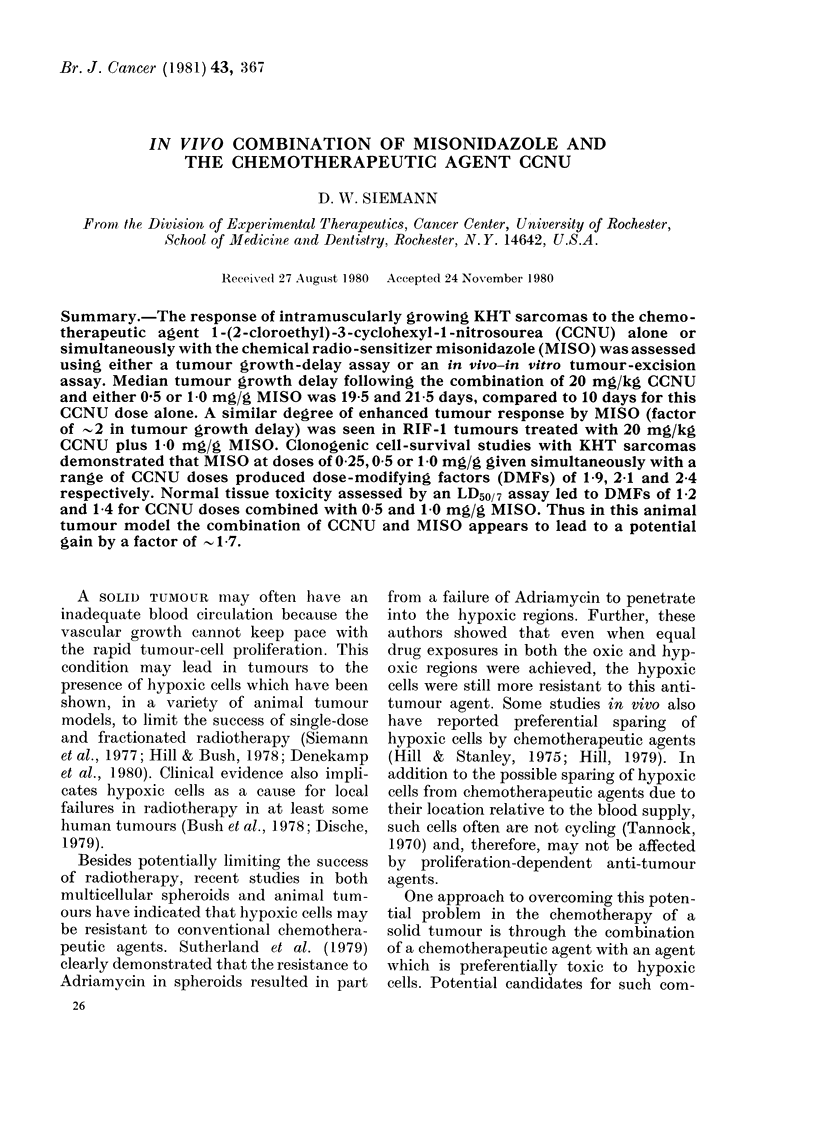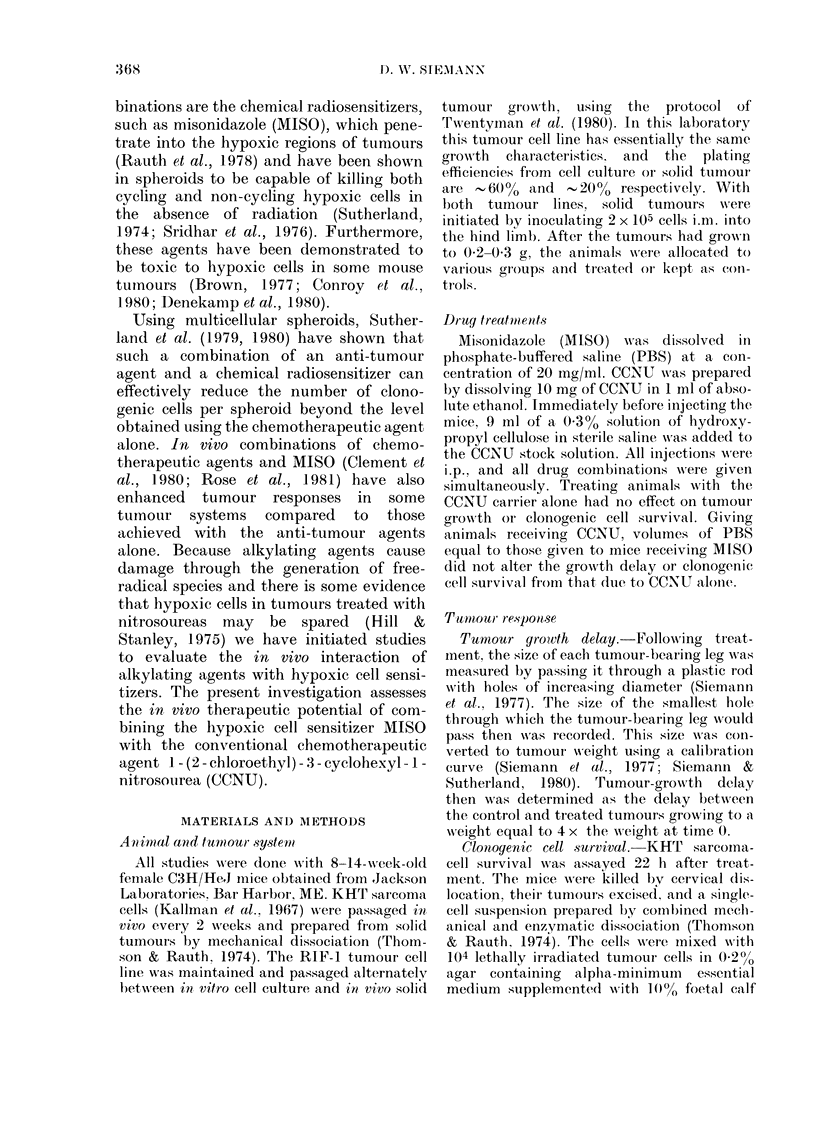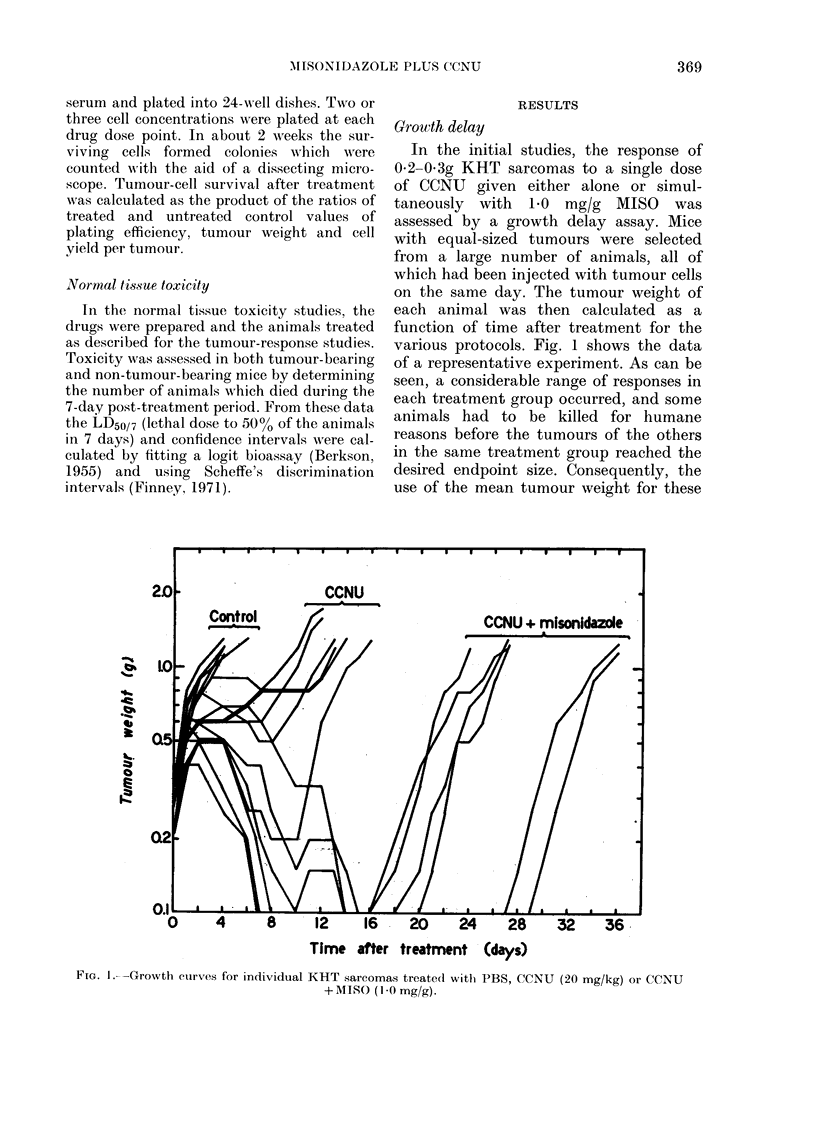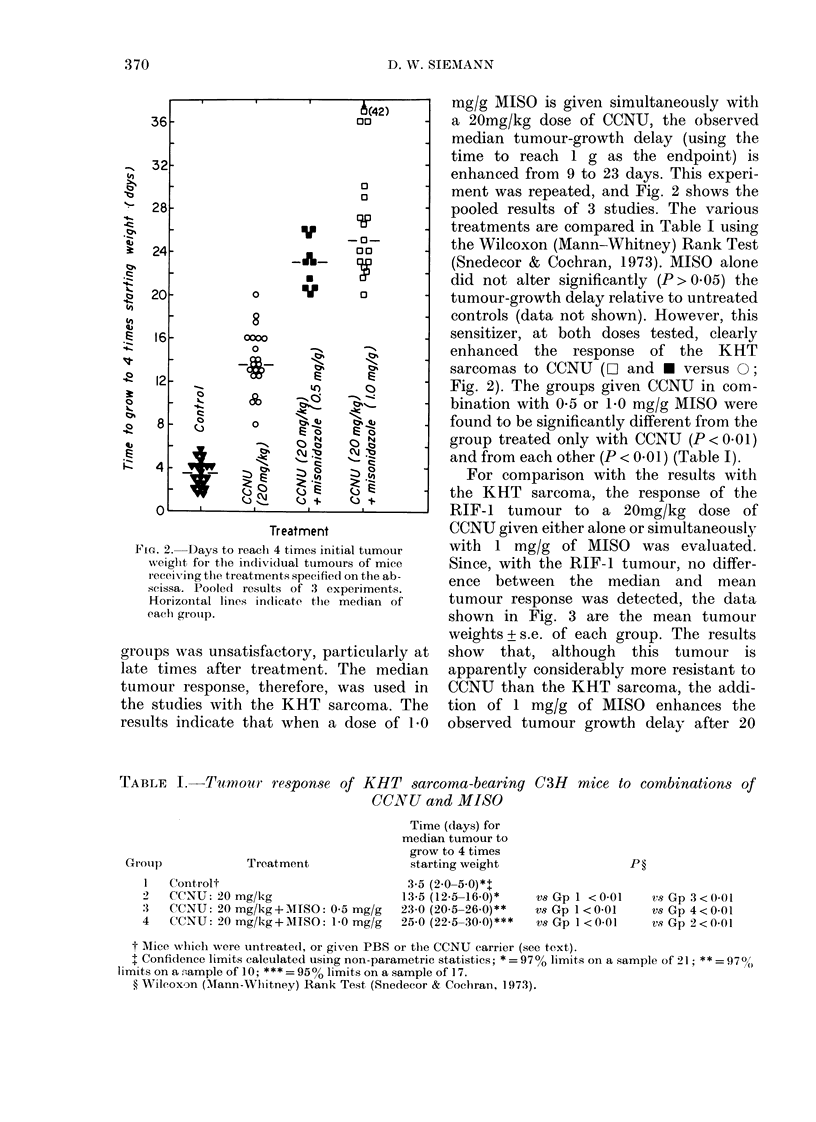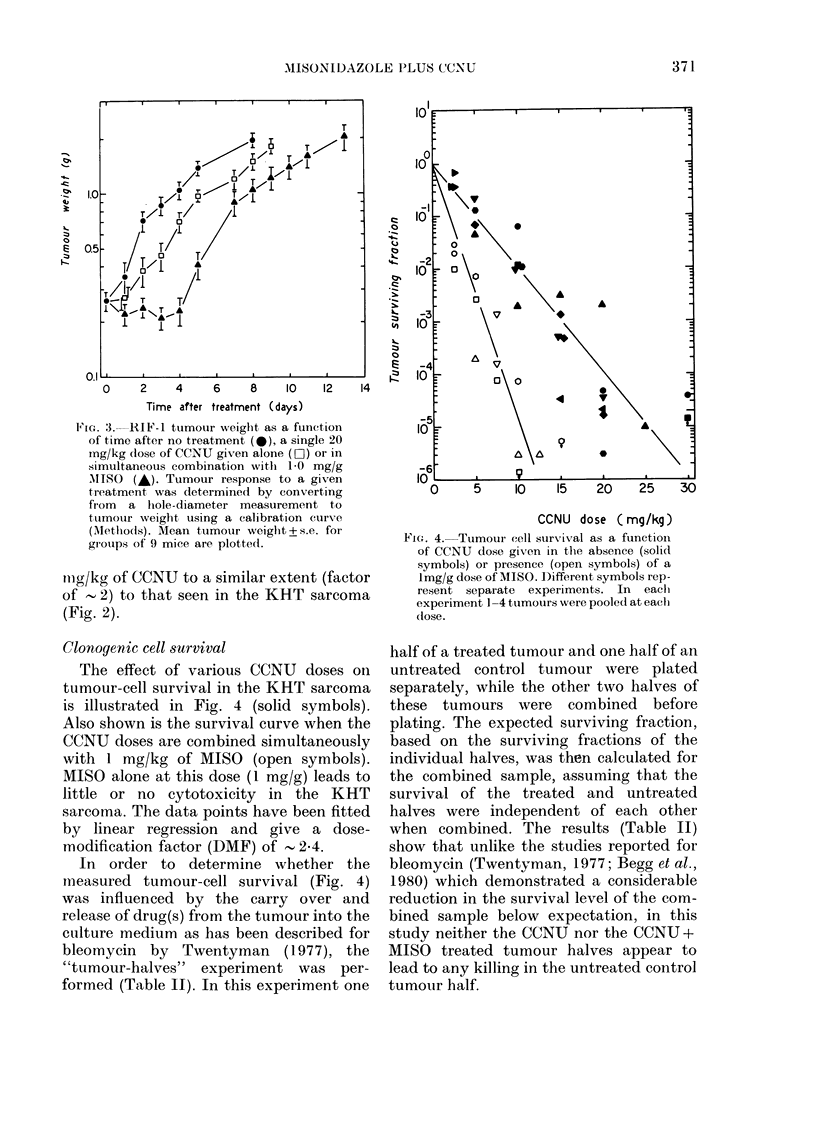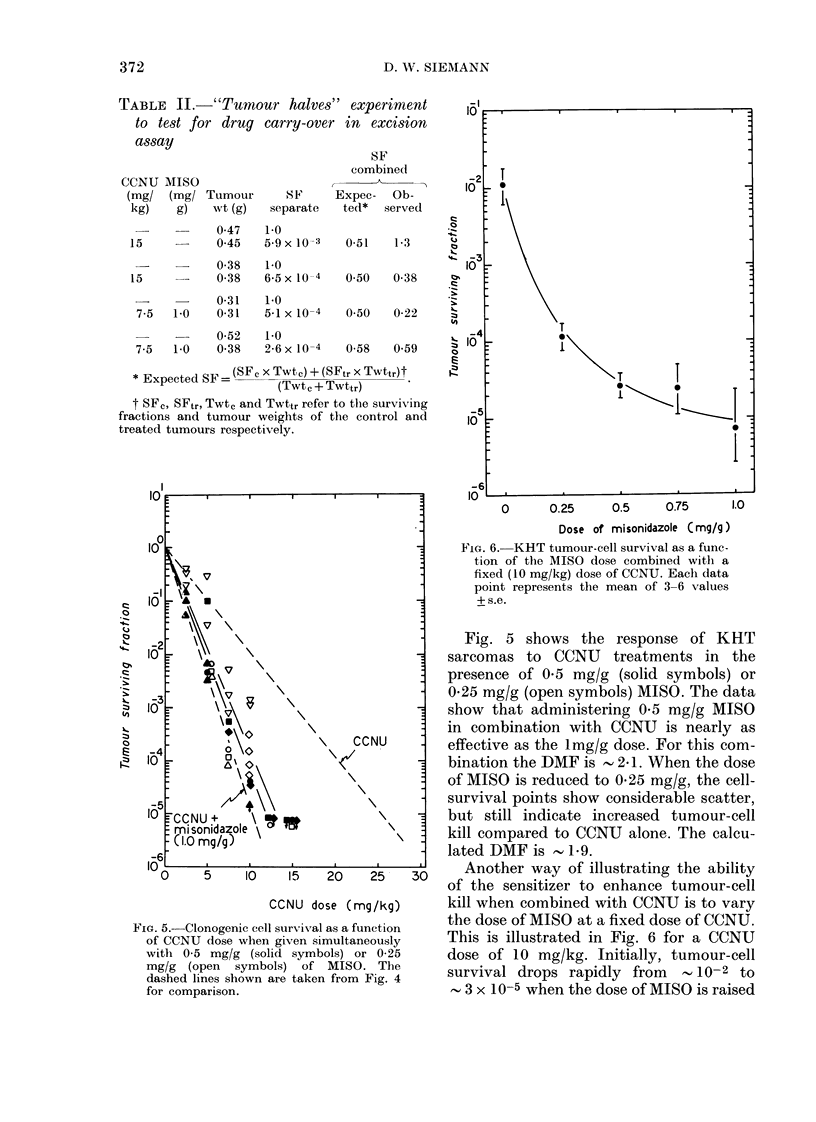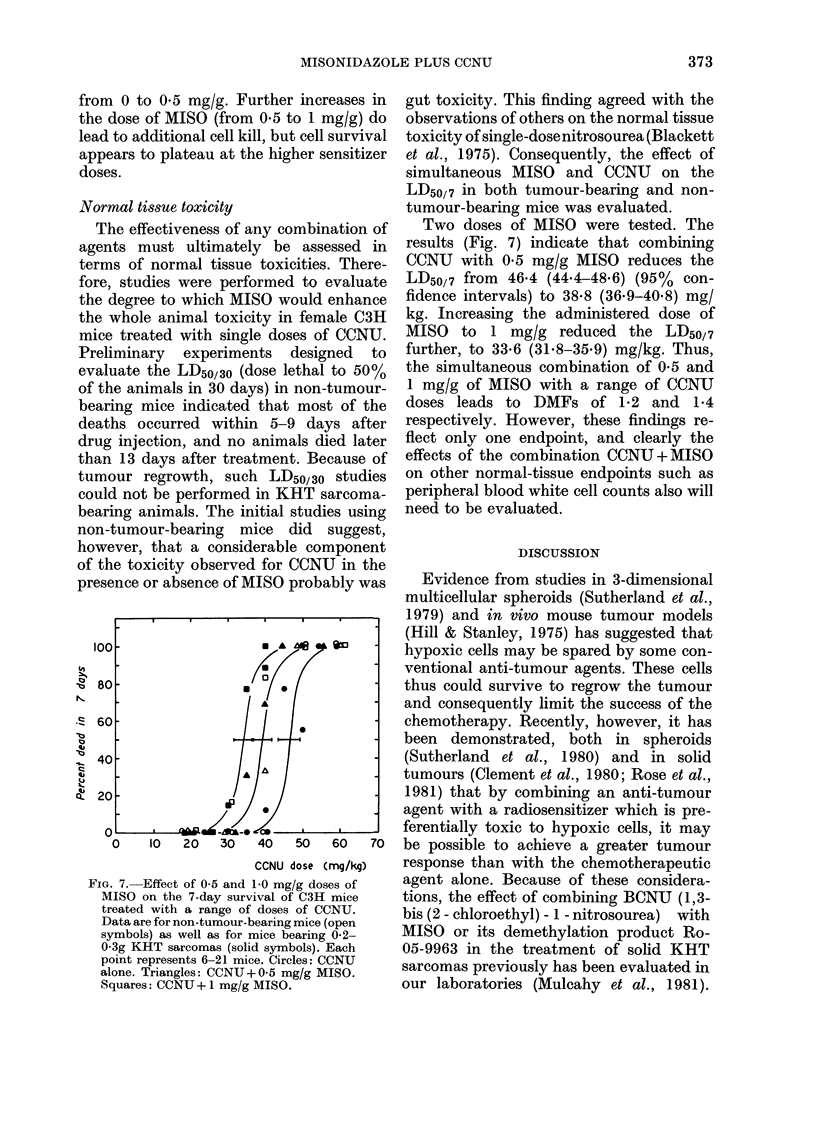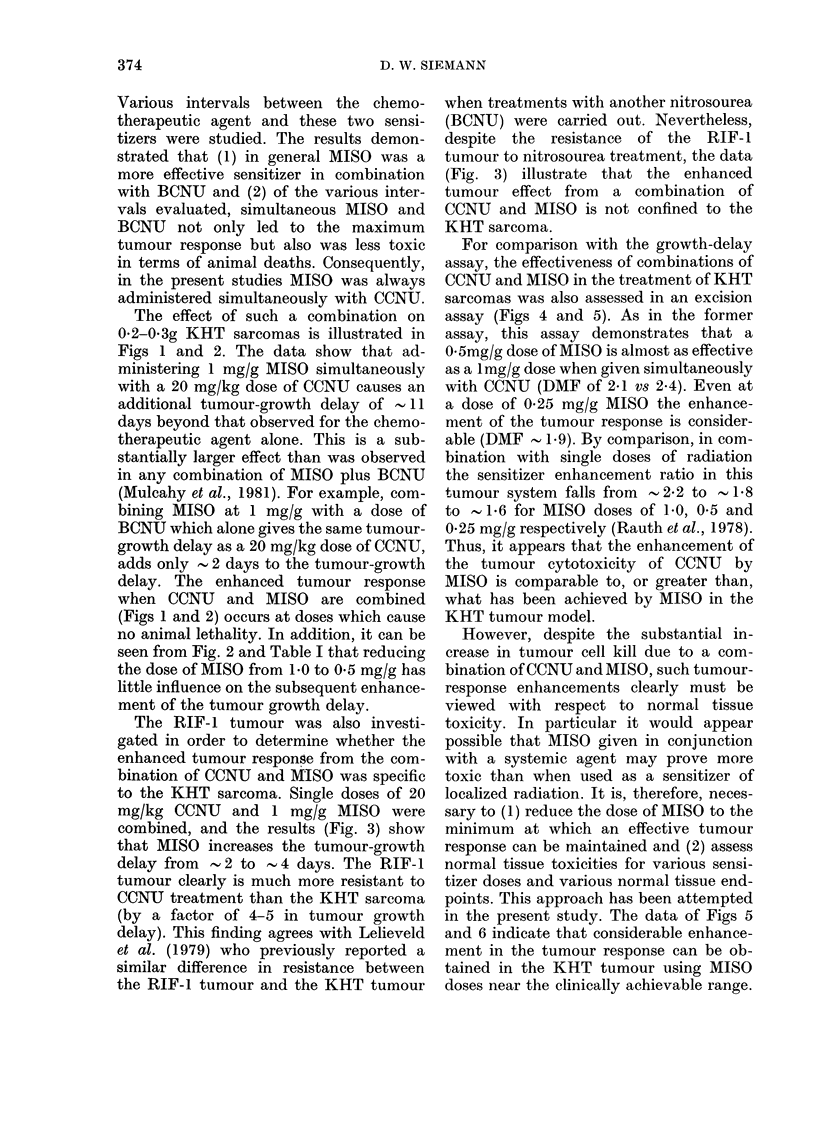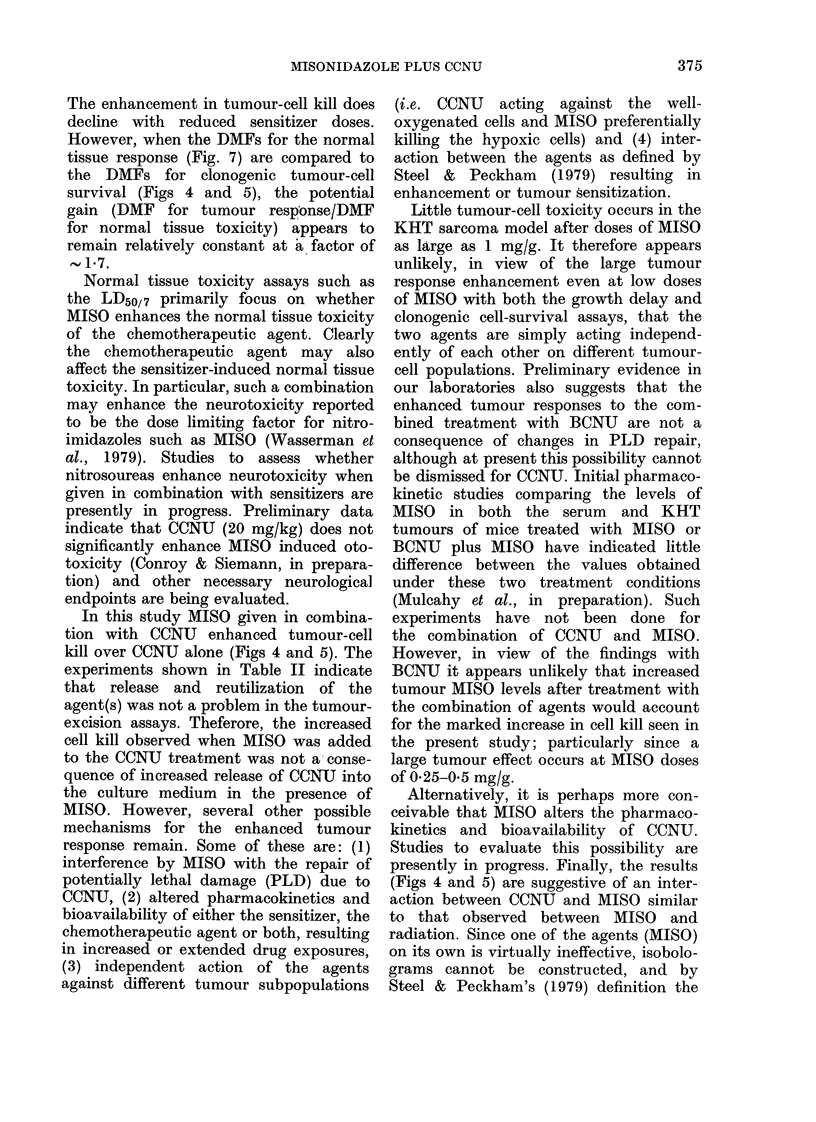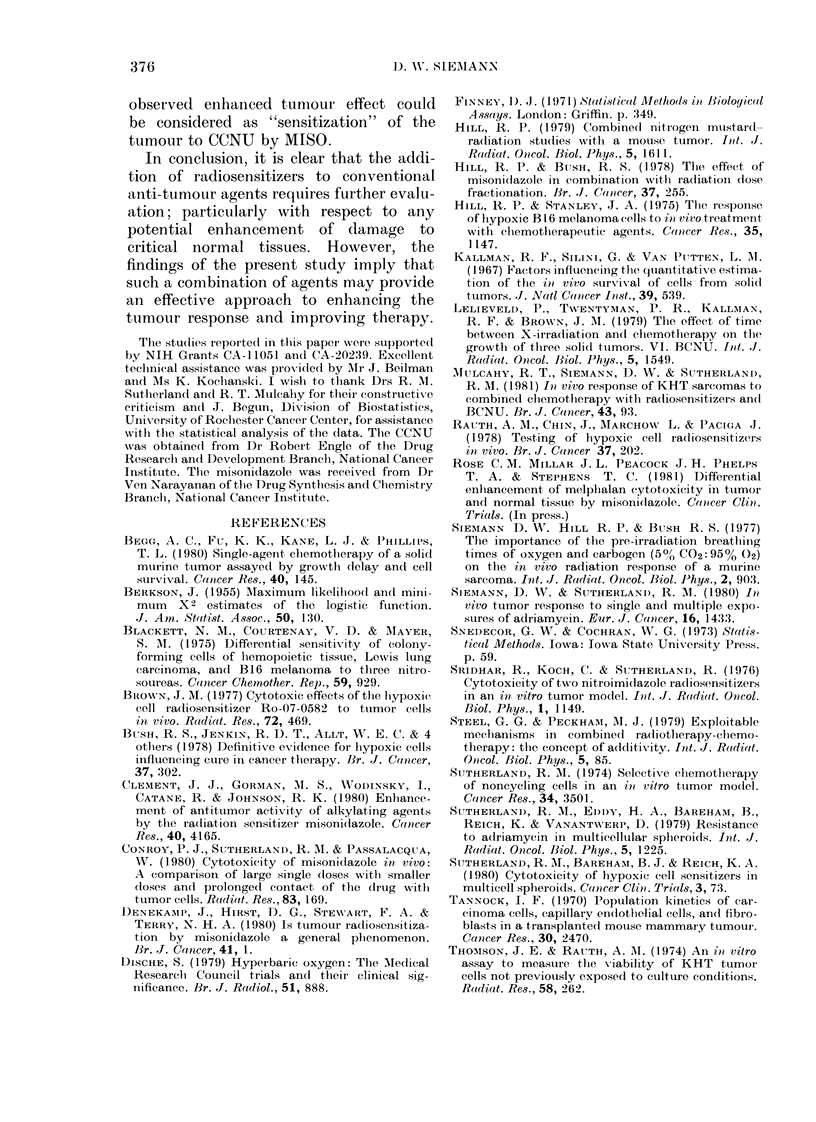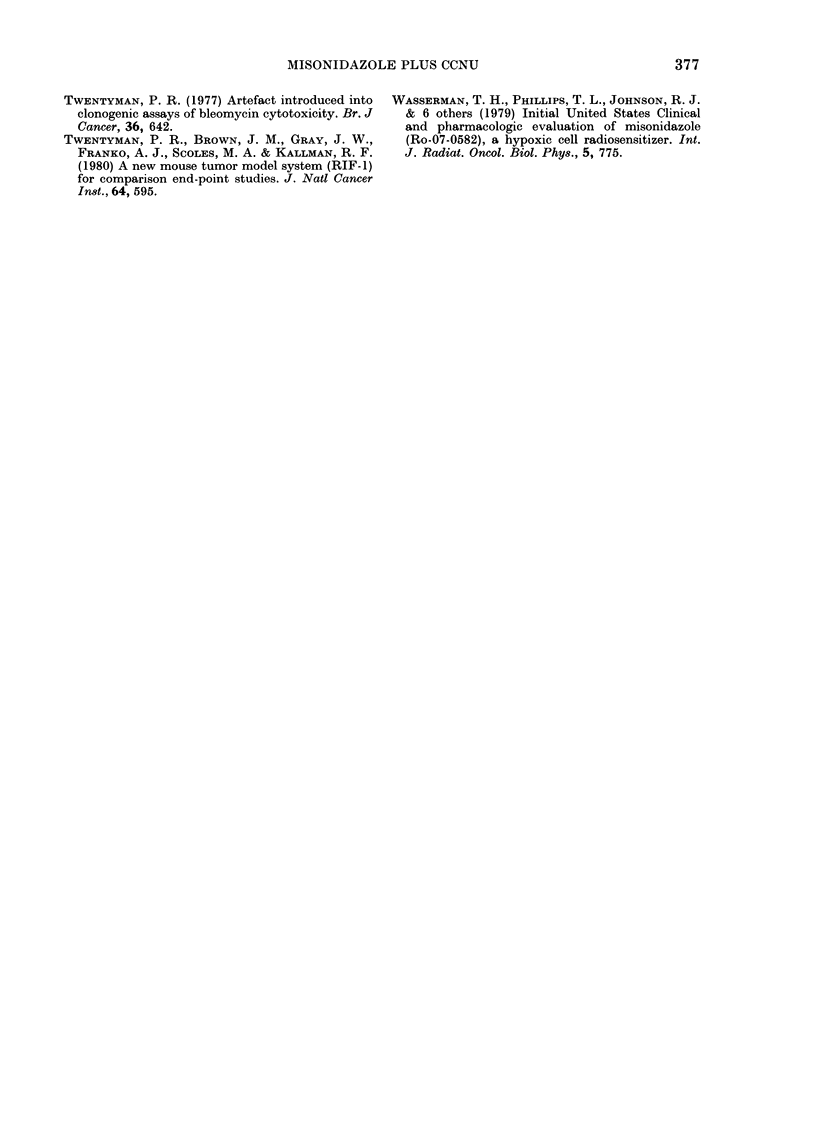# In vivo combination of misonidazole and the chemotherapeutic agent CCNU.

**DOI:** 10.1038/bjc.1981.57

**Published:** 1981-03

**Authors:** D. W. Siemann

## Abstract

The response of intramuscularly growing KHT sarcomas to the chemotherapeutic agent (1-(2-cloroethyl)-3-cyclohexyl-1-nitrosourea (CCNU) alone or simultaneously with the chemical radio-sensitizer misonidazole (MISO) was assessed using either a tumour growth-delay assay or an in vivo-in vitro tumour-excision assay. Median tumour growth delay following the combination of 20 mg/kg CCNU and either 0.5 or 1.0 mg/g MISO was 19.5 and 21.5 days, compared to 10 days for this CCNU dose alone. A similar degree of enhanced tumour response by MISO (factor of approximately 2 in tumour growth delay) was seen in RIF-1 tumours treated with 20 mg/kg CCNU plus 1.0 mg/g MISO. Clonogenic cell-survival studies with KHT sarcomas demonstrated that MISO at doses of 0.25, 0.5 or 1.0 mg/g given simultaneously with a range of CCNU doses produced dose-modifying factors (DMFs) of 1.9, 2.1 and 2.4 respectively. Normal tissue toxicity assessed by an LD50/7 assay led to DMFs of 1.2 and 1.4 for CCNU doses combined with 0.5 and 1.0 mg/g MISO. Thus in this animal tumour model the combination of CCNU and MISO appears to lead to a potential gain by a factor of approximately 1.7.


*Br. J. Cancer* (1981) **43**, 367

### *IN VIVO* COMBINATION OF MISONIDAZOLE AND THE CHEMOTHERAPEUTIC AGENT CCNU

D. W. SIEMANN

*From the Division of Experimental Therapeutics, Cancer Center, University of Rochester, School of Medicine and Dentistry, Rochester, N.Y. 14642, U.S.A.*

Received 27 August 1980 Accepted 24 November 1980

**Summary.—The response of intramuscularly growing KHT sarcomas to the chemotherapeutic agent 1-(2-cloroethyl)-3-cyclohexyl-1-nitrosourea (CCNU) alone or simultaneously with the chemical radio-sensitizer misonidazole (MISO) was assessed using either a tumour growth-delay assay or an *in vivo–in vitro* tumour-excision assay. Median tumour growth delay following the combination of 20 mg/kg CCNU and either 0·5 or 1·0 mg/g MISO was 19·5 and 21·5 days, compared to 10 days for this CCNU dose alone. A similar degree of enhanced tumour response by MISO (factor of ~2 in tumour growth delay) was seen in RIF-1 tumours treated with 20 mg/kg CCNU plus 1·0 mg/g MISO. Clonogenic cell-survival studies with KHT sarcomas demonstrated that MISO at doses of 0·25, 0·5 or 1·0 mg/g given simultaneously with a range of CCNU doses produced dose-modifying factors (DMFs) of 1·9, 2·1 and 2·4 respectively. Normal tissue toxicity assessed by an $LD_{50/7}$ assay led to DMFs of 1·2 and 1·4 for CCNU doses combined with 0·5 and 1·0 mg/g MISO. Thus in this animal tumour model the combination of CCNU and MISO appears to lead to a potential gain by a factor of ~1·7.**

A SOLID TUMOUR may often have an inadequate blood circulation because the vascular growth cannot keep pace with the rapid tumour-cell proliferation. This condition may lead in tumours to the presence of hypoxic cells which have been shown, in a variety of animal tumour models, to limit the success of single-dose and fractionated radiotherapy (Siemann *et al.*, 1977; Hill & Bush, 1978; Denekamp *et al.*, 1980). Clinical evidence also implicates hypoxic cells as a cause for local failures in radiotherapy in at least some human tumours (Bush *et al.*, 1978; Dische, 1979).

Besides potentially limiting the success of radiotherapy, recent studies in both multicellular spheroids and animal tumours have indicated that hypoxic cells may be resistant to conventional chemotherapeutic agents. Sutherland *et al.* (1979) clearly demonstrated that the resistance to Adriamycin in spheroids resulted in part from a failure of Adriamycin to penetrate into the hypoxic regions. Further, these authors showed that even when equal drug exposures in both the oxic and hypoxic regions were achieved, the hypoxic cells were still more resistant to this antitumour agent. Some studies *in vivo* also have reported preferential sparing of hypoxic cells by chemotherapeutic agents (Hill & Stanley, 1975; Hill, 1979). In addition to the possible sparing of hypoxic cells from chemotherapeutic agents due to their location relative to the blood supply, such cells often are not cycling (Tannock, 1970) and, therefore, may not be affected by proliferation-dependent anti-tumour agents.

One approach to overcoming this potential problem in the chemotherapy of a solid tumour is through the combination of a chemotherapeutic agent with an agent which is preferentially toxic to hypoxic cells. Potential candidates for such combinations are the chemical radiosensitizers, such as misonidazole (MISO), which penetrate into the hypoxic regions of tumours (Rauth *et al.*, 1978) and have been shown in spheroids to be capable of killing both cycling and non-cycling hypoxic cells in the absence of radiation (Sutherland, 1974; Sridhar *et al.*, 1976). Furthermore, these agents have been demonstrated to be toxic to hypoxic cells in some mouse tumours (Brown, 1977; Conroy *et al.*, 1980; Denekamp *et al.*, 1980).

Using multicellular spheroids, Sutherland *et al.* (1979, 1980) have shown that such a combination of an anti-tumour agent and a chemical radiosensitizer can effectively reduce the number of clonogenic cells per spheroid beyond the level obtained using the chemotherapeutic agent alone. *In vivo* combinations of chemotherapeutic agents and MISO (Clement *et al.*, 1980; Rose *et al.*, 1981) have also enhanced tumour responses in some tumour systems compared to those achieved with the anti-tumour agents alone. Because alkylating agents cause damage through the generation of free-radical species and there is some evidence that hypoxic cells in tumours treated with nitrosoureas may be spared (Hill & Stanley, 1975) we have initiated studies to evaluate the *in vivo* interaction of alkylating agents with hypoxic cell sensitizers. The present investigation assesses the *in vivo* therapeutic potential of combining the hypoxic cell sensitizer MISO with the conventional chemotherapeutic agent 1-(2-chloroethyl)-3-cyclohexyl-1-nitrosourea (CCNU).

### MATERIALS AND METHODS

### *Animal and tumour system*

All studies were done with 8–14-week-old female C3H/HeJ mice obtained from Jackson Laboratories, Bar Harbor, ME. KHT sarcoma cells (Kallman *et al.*, 1967) were passaged *in vivo* every 2 weeks and prepared from solid tumours by mechanical dissociation (Thomson & Rauth, 1974). The RIF-1 tumour cell line was maintained and passaged alternately between *in vitro* cell culture and *in vivo* solid tumour growth, using the protocol of Twentyman *et al.* (1980). In this laboratory this tumour cell line has essentially the same growth characteristics, and the plating efficiencies from cell culture or solid tumour are ~60% and ~20% respectively. With both tumour lines, solid tumours were initiated by inoculating $2 \times 10^5$ cells i.m. into the hind limb. After the tumours had grown to 0·2–0·3 g, the animals were allocated to various groups and treated or kept as controls.

### *Drug treatments*

Misonidazole (MISO) was dissolved in phosphate-buffered saline (PBS) at a concentration of 20 mg/ml. CCNU was prepared by dissolving 10 mg of CCNU in 1 ml of absolute ethanol. Immediately before injecting the mice, 9 ml of a 0·3% solution of hydroxypropyl cellulose in sterile saline was added to the CCNU stock solution. All injections were i.p., and all drug combinations were given simultaneously. Treating animals with the CCNU carrier alone had no effect on tumour growth or clonogenic cell survival. Giving animals receiving CCNU, volumes of PBS equal to those given to mice receiving MISO did not alter the growth delay or clonogenic cell survival from that due to CCNU alone.

### *Tumour response*

*Tumour growth delay.*—Following treatment, the size of each tumour-bearing leg was measured by passing it through a plastic rod with holes of increasing diameter (Siemann *et al.*, 1977). The size of the smallest hole through which the tumour-bearing leg would pass then was recorded. This size was converted to tumour weight using a calibration curve (Siemann *et al.*, 1977; Siemann & Sutherland, 1980). Tumour-growth delay then was determined as the delay between the control and treated tumours growing to a weight equal to 4× the weight at time 0.

*Clonogenic cell survival.*—KHT sarcoma-cell survival was assayed 22 h after treatment. The mice were killed by cervical dislocation, their tumours excised, and a single-cell suspension prepared by combined mechanical and enzymatic dissociation (Thomson & Rauth, 1974). The cells were mixed with $10^4$ lethally irradiated tumour cells in 0·2% agar containing alpha-minimum essential medium supplemented with 10% foetal calf serum and plated into 24-well dishes. Two or three cell concentrations were plated at each drug dose point. In about 2 weeks the surviving cells formed colonies which were counted with the aid of a dissecting microscope. Tumour-cell survival after treatment was calculated as the product of the ratios of treated and untreated control values of plating efficiency, tumour weight and cell yield per tumour.

### *Normal tissue toxicity*

In the normal tissue toxicity studies, the drugs were prepared and the animals treated as described for the tumour-response studies. Toxicity was assessed in both tumour-bearing and non-tumour-bearing mice by determining the number of animals which died during the 7-day post-treatment period. From these data the $LD_{50/7}$ (lethal dose to 50% of the animals in 7 days) and confidence intervals were calculated by fitting a logit bioassay (Berkson, 1955) and using Scheffe's discrimination intervals (Finney, 1971).

### RESULTS

### *Growth delay*

In the initial studies, the response of 0·2–0·3g KHT sarcomas to a single dose of CCNU given either alone or simultaneously with 1·0 mg/g MISO was assessed by a growth delay assay. Mice with equal-sized tumours were selected from a large number of animals, all of which had been injected with tumour cells on the same day. The tumour weight of each animal was then calculated as a function of time after treatment for the various protocols. Fig. 1 shows the data of a representative experiment. As can be seen, a considerable range of responses in each treatment group occurred, and some animals had to be killed for humane reasons before the tumours of the others in the same treatment group reached the desired endpoint size. Consequently, the use of the mean tumour weight for these

FIG. 1.—Growth curves for individual KHT sarcomas treated with PBS, CCNU (20 mg/kg) or CCNU +MISO (1·0 mg/g).

FIG. 2.—Days to reach 4 times initial tumour weight for the individual tumours of mice receiving the treatments specified on the abscissa. Pooled results of 3 experiments. Horizontal lines indicate the median of each group.

groups was unsatisfactory, particularly at late times after treatment. The median tumour response, therefore, was used in the studies with the KHT sarcoma. The results indicate that when a dose of 1·0 mg/g MISO is given simultaneously with a 20mg/kg dose of CCNU, the observed median tumour-growth delay (using the time to reach 1 g as the endpoint) is enhanced from 9 to 23 days. This experiment was repeated, and Fig. 2 shows the pooled results of 3 studies. The various treatments are compared in Table I using the Wilcoxon (Mann–Whitney) Rank Test (Snedecor & Cochran, 1973). MISO alone did not alter significantly ($P > 0{\cdot}05$) the tumour-growth delay relative to untreated controls (data not shown). However, this sensitizer, at both doses tested, clearly enhanced the response of the KHT sarcomas to CCNU (□ and ■ versus ○; Fig. 2). The groups given CCNU in combination with 0·5 or 1·0 mg/g MISO were found to be significantly different from the group treated only with CCNU ($P < 0{\cdot}01$) and from each other ($P < 0{\cdot}01$) (Table I).

For comparison with the results with the KHT sarcoma, the response of the RIF-1 tumour to a 20mg/kg dose of CCNU given either alone or simultaneously with 1 mg/g of MISO was evaluated. Since, with the RIF-1 tumour, no difference between the median and mean tumour response was detected, the data shown in Fig. 3 are the mean tumour weights ± s.e. of each group. The results show that, although this tumour is apparently considerably more resistant to CCNU than the KHT sarcoma, the addition of 1 mg/g of MISO enhances the observed tumour growth delay after 20

TABLE I.—*Tumour response of KHT sarcoma-bearing C3H mice to combinations of CCNU and MISO*

| Group | Treatment | Time (days) for median tumour to grow to 4 times starting weight | P§ | |
|---|---|---|---|---|
| 1 | Control† | 3·5 (2·0–5·0)*‡ | | |
| 2 | CCNU: 20 mg/kg | 13·5 (12·5–16·0)* | *vs* Gp 1 < 0·01 | *vs* Gp 3 < 0·01 |
| 3 | CCNU: 20 mg/kg + MISO: 0·5 mg/g | 23·0 (20·5–26·0)** | *vs* Gp 1 < 0·01 | *vs* Gp 4 < 0·01 |
| 4 | CCNU: 20 mg/kg + MISO: 1·0 mg/g | 25·0 (22·5–30·0)*** | *vs* Gp 1 < 0·01 | *vs* Gp 2 < 0·01 |

† Mice which were untreated, or given PBS or the CCNU carrier (see text).
‡ Confidence limits calculated using non-parametric statistics; * = 97% limits on a sample of 21; ** = 97% limits on a sample of 10; *** = 95% limits on a sample of 17.
§ Wilcoxon (Mann-Whitney) Rank Test (Snedecor & Cochran, 1973).

FIG. 3.—RIF-1 tumour weight as a function of time after no treatment (●), a single 20 mg/kg dose of CCNU given alone (□) or in simultaneous combination with 1·0 mg/g MISO (▲). Tumour response to a given treatment was determined by converting from a hole-diameter measurement to tumour weight using a calibration curve (Methods). Mean tumour weight ± s.e. for groups of 9 mice are plotted.

FIG. 4.—Tumour cell survival as a function of CCNU dose given in the absence (solid symbols) or presence (open symbols) of a 1mg/g dose of MISO. Different symbols represent separate experiments. In each experiment 1–4 tumours were pooled at each dose.

mg/kg of CCNU to a similar extent (factor of ~2) to that seen in the KHT sarcoma (Fig. 2).

*Clonogenic cell survival*

The effect of various CCNU doses on tumour-cell survival in the KHT sarcoma is illustrated in Fig. 4 (solid symbols). Also shown is the survival curve when the CCNU doses are combined simultaneously with 1 mg/kg of MISO (open symbols). MISO alone at this dose (1 mg/g) leads to little or no cytotoxicity in the KHT sarcoma. The data points have been fitted by linear regression and give a dose-modification factor (DMF) of ~2·4.

In order to determine whether the measured tumour-cell survival (Fig. 4) was influenced by the carry over and release of drug(s) from the tumour into the culture medium as has been described for bleomycin by Twentyman (1977), the "tumour-halves" experiment was performed (Table II). In this experiment one half of a treated tumour and one half of an untreated control tumour were plated separately, while the other two halves of these tumours were combined before plating. The expected surviving fraction, based on the surviving fractions of the individual halves, was then calculated for the combined sample, assuming that the survival of the treated and untreated halves were independent of each other when combined. The results (Table II) show that unlike the studies reported for bleomycin (Twentyman, 1977; Begg *et al.*, 1980) which demonstrated a considerable reduction in the survival level of the combined sample below expectation, in this study neither the CCNU nor the CCNU + MISO treated tumour halves appear to lead to any killing in the untreated control tumour half.

TABLE II.—*"Tumour halves" experiment to test for drug carry-over in excision assay*

| CCNU (mg/kg) | MISO (mg/g) | Tumour wt (g) | SF separate | SF combined Expected* | Observed |
|---|---|---|---|---|---|
| — | — | 0·47 | 1·0 | | |
| 15 | — | 0·45 | $5{\cdot}9\times10^{-3}$ | 0·51 | 1·3 |
| — | — | 0·38 | 1·0 | | |
| 15 | — | 0·38 | $6{\cdot}5\times10^{-4}$ | 0·50 | 0·38 |
| — | — | 0·31 | 1·0 | | |
| 7·5 | 1·0 | 0·31 | $5{\cdot}1\times10^{-4}$ | 0·50 | 0·22 |
| — | — | 0·52 | 1·0 | | |
| 7·5 | 1·0 | 0·38 | $2{\cdot}6\times10^{-4}$ | 0·58 | 0·59 |

$$\text{* Expected SF} = \frac{(SF_c \times Twt_c) + (SF_{tr} \times Twt_{tr})\dagger}{(Twt_c + Twt_{tr})}.$$

† $SF_c$, $SF_{tr}$, $Twt_c$ and $Twt_{tr}$ refer to the surviving fractions and tumour weights of the control and treated tumours respectively.

FIG. 5.—Clonogenic cell survival as a function of CCNU dose when given simultaneously with 0·5 mg/g (solid symbols) or 0·25 mg/g (open symbols) of MISO. The dashed lines shown are taken from Fig. 4 for comparison.

FIG. 6.—KHT tumour-cell survival as a function of the MISO dose combined with a fixed (10 mg/kg) dose of CCNU. Each data point represents the mean of 3–6 values ±s.e.

Fig. 5 shows the response of KHT sarcomas to CCNU treatments in the presence of 0·5 mg/g (solid symbols) or 0·25 mg/g (open symbols) MISO. The data show that administering 0·5 mg/g MISO in combination with CCNU is nearly as effective as the 1mg/g dose. For this combination the DMF is ~2·1. When the dose of MISO is reduced to 0·25 mg/g, the cell-survival points show considerable scatter, but still indicate increased tumour-cell kill compared to CCNU alone. The calculated DMF is ~1·9.

Another way of illustrating the ability of the sensitizer to enhance tumour-cell kill when combined with CCNU is to vary the dose of MISO at a fixed dose of CCNU. This is illustrated in Fig. 6 for a CCNU dose of 10 mg/kg. Initially, tumour-cell survival drops rapidly from $\sim10^{-2}$ to $\sim3\times10^{-5}$ when the dose of MISO is raised from 0 to 0·5 mg/g. Further increases in the dose of MISO (from 0·5 to 1 mg/g) do lead to additional cell kill, but cell survival appears to plateau at the higher sensitizer doses.

*Normal tissue toxicity*

The effectiveness of any combination of agents must ultimately be assessed in terms of normal tissue toxicities. Therefore, studies were performed to evaluate the degree to which MISO would enhance the whole animal toxicity in female C3H mice treated with single doses of CCNU. Preliminary experiments designed to evaluate the $LD_{50/30}$ (dose lethal to 50% of the animals in 30 days) in non-tumour-bearing mice indicated that most of the deaths occurred within 5–9 days after drug injection, and no animals died later than 13 days after treatment. Because of tumour regrowth, such $LD_{50/30}$ studies could not be performed in KHT sarcoma-bearing animals. The initial studies using non-tumour-bearing mice did suggest, however, that a considerable component of the toxicity observed for CCNU in the presence or absence of MISO probably was gut toxicity. This finding agreed with the observations of others on the normal tissue toxicity of single-dose nitrosourea (Blackett *et al.*, 1975). Consequently, the effect of simultaneous MISO and CCNU on the $LD_{50/7}$ in both tumour-bearing and non-tumour-bearing mice was evaluated.

FIG. 7.—Effect of 0·5 and 1·0 mg/g doses of MISO on the 7-day survival of C3H mice treated with a range of doses of CCNU. Data are for non-tumour-bearing mice (open symbols) as well as for mice bearing 0·2–0·3g KHT sarcomas (solid symbols). Each point represents 6–21 mice. Circles: CCNU alone. Triangles: CCNU + 0·5 mg/g MISO. Squares: CCNU + 1 mg/g MISO.

Two doses of MISO were tested. The results (Fig. 7) indicate that combining CCNU with 0·5 mg/g MISO reduces the $LD_{50/7}$ from 46·4 (44·4–48·6) (95% confidence intervals) to 38·8 (36·9–40·8) mg/kg. Increasing the administered dose of MISO to 1 mg/g reduced the $LD_{50/7}$ further, to 33·6 (31·8–35·9) mg/kg. Thus, the simultaneous combination of 0·5 and 1 mg/g of MISO with a range of CCNU doses leads to DMFs of 1·2 and 1·4 respectively. However, these findings reflect only one endpoint, and clearly the effects of the combination CCNU + MISO on other normal-tissue endpoints such as peripheral blood white cell counts also will need to be evaluated.

### DISCUSSION

Evidence from studies in 3-dimensional multicellular spheroids (Sutherland *et al.*, 1979) and *in vivo* mouse tumour models (Hill & Stanley, 1975) has suggested that hypoxic cells may be spared by some conventional anti-tumour agents. These cells thus could survive to regrow the tumour and consequently limit the success of the chemotherapy. Recently, however, it has been demonstrated, both in spheroids (Sutherland *et al.*, 1980) and in solid tumours (Clement *et al.*, 1980; Rose *et al.*, 1981) that by combining an anti-tumour agent with a radiosensitizer which is preferentially toxic to hypoxic cells, it may be possible to achieve a greater tumour response than with the chemotherapeutic agent alone. Because of these considerations, the effect of combining BCNU (1,3-bis (2 - chloroethyl) - 1 - nitrosourea) with MISO or its demethylation product Ro-05-9963 in the treatment of solid KHT sarcomas previously has been evaluated in our laboratories (Mulcahy *et al.*, 1981).

Various intervals between the chemotherapeutic agent and these two sensitizers were studied. The results demonstrated that (1) in general MISO was a more effective sensitizer in combination with BCNU and (2) of the various intervals evaluated, simultaneous MISO and BCNU not only led to the maximum tumour response but also was less toxic in terms of animal deaths. Consequently, in the present studies MISO was always administered simultaneously with CCNU.

The effect of such a combination on 0·2–0·3g KHT sarcomas is illustrated in Figs 1 and 2. The data show that administering 1 mg/g MISO simultaneously with a 20 mg/kg dose of CCNU causes an additional tumour-growth delay of ~11 days beyond that observed for the chemotherapeutic agent alone. This is a substantially larger effect than was observed in any combination of MISO plus BCNU (Mulcahy *et al.*, 1981). For example, combining MISO at 1 mg/g with a dose of BCNU which alone gives the same tumour-growth delay as a 20 mg/kg dose of CCNU, adds only ~2 days to the tumour-growth delay. The enhanced tumour response when CCNU and MISO are combined (Figs 1 and 2) occurs at doses which cause no animal lethality. In addition, it can be seen from Fig. 2 and Table I that reducing the dose of MISO from 1·0 to 0·5 mg/g has little influence on the subsequent enhancement of the tumour growth delay.

The RIF-1 tumour was also investigated in order to determine whether the enhanced tumour response from the combination of CCNU and MISO was specific to the KHT sarcoma. Single doses of 20 mg/kg CCNU and 1 mg/g MISO were combined, and the results (Fig. 3) show that MISO increases the tumour-growth delay from ~2 to ~4 days. The RIF-1 tumour clearly is much more resistant to CCNU treatment than the KHT sarcoma (by a factor of 4–5 in tumour growth delay). This finding agrees with Lelieveld *et al.* (1979) who previously reported a similar difference in resistance between the RIF-1 tumour and the KHT tumour when treatments with another nitrosourea (BCNU) were carried out. Nevertheless, despite the resistance of the RIF-1 tumour to nitrosourea treatment, the data (Fig. 3) illustrate that the enhanced tumour effect from a combination of CCNU and MISO is not confined to the KHT sarcoma.

For comparison with the growth-delay assay, the effectiveness of combinations of CCNU and MISO in the treatment of KHT sarcomas was also assessed in an excision assay (Figs 4 and 5). As in the former assay, this assay demonstrates that a 0·5mg/g dose of MISO is almost as effective as a 1mg/g dose when given simultaneously with CCNU (DMF of 2·1 *vs* 2·4). Even at a dose of 0·25 mg/g MISO the enhancement of the tumour response is considerable (DMF ~1·9). By comparison, in combination with single doses of radiation the sensitizer enhancement ratio in this tumour system falls from ~2·2 to ~1·8 to ~1·6 for MISO doses of 1·0, 0·5 and 0·25 mg/g respectively (Rauth *et al.*, 1978). Thus, it appears that the enhancement of the tumour cytotoxicity of CCNU by MISO is comparable to, or greater than, what has been achieved by MISO in the KHT tumour model.

However, despite the substantial increase in tumour cell kill due to a combination of CCNU and MISO, such tumour-response enhancements clearly must be viewed with respect to normal tissue toxicity. In particular it would appear possible that MISO given in conjunction with a systemic agent may prove more toxic than when used as a sensitizer of localized radiation. It is, therefore, necessary to (1) reduce the dose of MISO to the minimum at which an effective tumour response can be maintained and (2) assess normal tissue toxicities for various sensitizer doses and various normal tissue end-points. This approach has been attempted in the present study. The data of Figs 5 and 6 indicate that considerable enhancement in the tumour response can be obtained in the KHT tumour using MISO doses near the clinically achievable range.

The enhancement in tumour-cell kill does decline with reduced sensitizer doses. However, when the DMFs for the normal tissue response (Fig. 7) are compared to the DMFs for clonogenic tumour-cell survival (Figs 4 and 5), the potential gain (DMF for tumour response/DMF for normal tissue toxicity) appears to remain relatively constant at a factor of ~1·7.

Normal tissue toxicity assays such as the $LD_{50/7}$ primarily focus on whether MISO enhances the normal tissue toxicity of the chemotherapeutic agent. Clearly the chemotherapeutic agent may also affect the sensitizer-induced normal tissue toxicity. In particular, such a combination may enhance the neurotoxicity reported to be the dose limiting factor for nitroimidazoles such as MISO (Wasserman *et al.*, 1979). Studies to assess whether nitrosoureas enhance neurotoxicity when given in combination with sensitizers are presently in progress. Preliminary data indicate that CCNU (20 mg/kg) does not significantly enhance MISO induced ototoxicity (Conroy & Siemann, in preparation) and other necessary neurological endpoints are being evaluated.

In this study MISO given in combination with CCNU enhanced tumour-cell kill over CCNU alone (Figs 4 and 5). The experiments shown in Table II indicate that release and reutilization of the agent(s) was not a problem in the tumour-excision assays. Theferore, the increased cell kill observed when MISO was added to the CCNU treatment was not a consequence of increased release of CCNU into the culture medium in the presence of MISO. However, several other possible mechanisms for the enhanced tumour response remain. Some of these are: (1) interference by MISO with the repair of potentially lethal damage (PLD) due to CCNU, (2) altered pharmacokinetics and bioavailability of either the sensitizer, the chemotherapeutic agent or both, resulting in increased or extended drug exposures, (3) independent action of the agents against different tumour subpopulations (*i.e.* CCNU acting against the well-oxygenated cells and MISO preferentially killing the hypoxic cells) and (4) interaction between the agents as defined by Steel & Peckham (1979) resulting in enhancement or tumour sensitization.

Little tumour-cell toxicity occurs in the KHT sarcoma model after doses of MISO as large as 1 mg/g. It therefore appears unlikely, in view of the large tumour response enhancement even at low doses of MISO with both the growth delay and clonogenic cell-survival assays, that the two agents are simply acting independently of each other on different tumour-cell populations. Preliminary evidence in our laboratories also suggests that the enhanced tumour responses to the combined treatment with BCNU are not a consequence of changes in PLD repair, although at present this possibility cannot be dismissed for CCNU. Initial pharmacokinetic studies comparing the levels of MISO in both the serum and KHT tumours of mice treated with MISO or BCNU plus MISO have indicated little difference between the values obtained under these two treatment conditions (Mulcahy *et al.*, in preparation). Such experiments have not been done for the combination of CCNU and MISO. However, in view of the findings with BCNU it appears unlikely that increased tumour MISO levels after treatment with the combination of agents would account for the marked increase in cell kill seen in the present study; particularly since a large tumour effect occurs at MISO doses of 0·25–0·5 mg/g.

Alternatively, it is perhaps more conceivable that MISO alters the pharmacokinetics and bioavailability of CCNU. Studies to evaluate this possibility are presently in progress. Finally, the results (Figs 4 and 5) are suggestive of an interaction between CCNU and MISO similar to that observed between MISO and radiation. Since one of the agents (MISO) on its own is virtually ineffective, isobolograms cannot be constructed, and by Steel & Peckham's (1979) definition the observed enhanced tumour effect could be considered as "sensitization" of the tumour to CCNU by MISO.

In conclusion, it is clear that the addition of radiosensitizers to conventional anti-tumour agents requires further evaluation; particularly with respect to any potential enhancement of damage to critical normal tissues. However, the findings of the present study imply that such a combination of agents may provide an effective approach to enhancing the tumour response and improving therapy.

The studies reported in this paper were supported by NIH Grants CA-11051 and CA-20239. Excellent technical assistance was provided by Mr J. Beilman and Ms K. Kochanski. I wish to thank Drs R. M. Sutherland and R. T. Mulcahy for their constructive criticism and J. Begun, Division of Biostatistics, University of Rochester Cancer Center, for assistance with the statistical analysis of the data. The CCNU was obtained from Dr Robert Engle of the Drug Research and Development Branch, National Cancer Institute. The misonidazole was received from Dr Ven Narayanan of the Drug Synthesis and Chemistry Branch, National Cancer Institute.

### REFERENCES

BEGG, A. C., FU, K. K., KANE, L. J. & PHILLIPS, T. L. (1980) Single-agent chemotherapy of a solid murine tumor assayed by growth delay and cell survival. *Cancer Res.*, **40**, 145.

BERKSON, J. (1955) Maximum likelihood and minimum $X^2$ estimates of the logistic function. *J. Am. Statist. Assoc.*, **50**, 130.

BLACKETT, N. M., COURTENAY, V. D. & MAYER, S. M. (1975) Differential sensitivity of colony-forming cells of hemopoietic tissue, Lewis lung carcinoma, and B16 melanoma to three nitrosoureas. *Cancer Chemother. Rep.*, **59**, 929.

BROWN, J. M. (1977) Cytotoxic effects of the hypoxic cell radiosensitizer Ro-07-0582 to tumor cells *in vivo*. *Radiat. Res.*, **72**, 469.

BUSH, R. S., JENKIN, R. D. T., ALLT, W. E. C. & 4 others (1978) Definitive evidence for hypoxic cells influencing cure in cancer therapy. *Br. J. Cancer*, **37**, 302.

CLEMENT, J. J., GORMAN, M. S., WODINSKY, I., CATANE, R. & JOHNSON, R. K. (1980) Enhancement of antitumor activity of alkylating agents by the radiation sensitizer misonidazole. *Cancer Res.*, **40**, 4165.

CONROY, P. J., SUTHERLAND, R. M. & PASSALACQUA, W. (1980) Cytotoxicity of misonidazole *in vivo*: A comparison of large single doses with smaller doses and prolonged contact of the drug with tumor cells. *Radiat. Res.*, **83**, 169.

DENEKAMP, J., HIRST, D. G., STEWART, F. A. & TERRY, N. H. A. (1980) Is tumour radiosensitization by misonidazole a general phenomenon. *Br. J. Cancer*, **41**, 1.

DISCHE, S. (1979) Hyperbaric oxygen: The Medical Research Council trials and their clinical significance. *Br. J. Radiol.*, **51**, 888.

FINNEY, D. J. (1971) *Statistical Methods in Biological Assays*. London: Griffin. p. 349.

HILL, R. P. (1979) Combined nitrogen mustard–radiation studies with a mouse tumor. *Int. J. Radiat. Oncol. Biol. Phys.*, **5**, 1611.

HILL, R. P. & BUSH, R. S. (1978) The effect of misonidazole in combination with radiation dose fractionation. *Br. J. Cancer*, **37**, 255.

HILL, R. P. & STANLEY, J. A. (1975) The response of hypoxic B16 melanoma cells to *in vivo* treatment with chemotherapeutic agents. *Cancer Res.*, **35**, 1147.

KALLMAN, R. F., SILINI, G. & VAN PUTTEN, L. M. (1967) Factors influencing the quantitative estimation of the *in vivo* survival of cells from solid tumors. *J. Natl Cancer Inst.*, **39**, 539.

LELIEVELD, P., TWENTYMAN, P. R., KALLMAN, R. F. & BROWN, J. M. (1979) The effect of time between X-irradiation and chemotherapy on the growth of three solid tumors. VI. BCNU. *Int. J. Radiat. Oncol. Biol. Phys.*, **5**, 1549.

MULCAHY, R. T., SIEMANN, D. W. & SUTHERLAND, R. M. (1981) *In vivo* response of KHT sarcomas to combined chemotherapy with radiosensitizers and BCNU. *Br. J. Cancer*, **43**, 93.

RAUTH, A. M., CHIN, J., MARCHOW L. & PACIGA J. (1978) Testing of hypoxic cell radiosensitizers *in vivo*. *Br. J. Cancer* **37**, 202.

ROSE C. M. MILLAR J. L. PEACOCK J. H. PHELPS T. A. & STEPHENS T. C. (1981) Differential enhancement of melphalan cytotoxicity in tumor and normal tissue by misonidazole. *Cancer Clin. Trials*. (In press.)

SIEMANN D. W. HILL R. P. & BUSH R. S. (1977) The importance of the pre-irradiation breathing times of oxygen and carbogen (5% $CO_2$:95% $O_2$) on the *in vivo* radiation response of a murine sarcoma. *Int. J. Radiat. Oncol. Biol. Phys.*, **2**, 903.

SIEMANN, D. W. & SUTHERLAND, R. M. (1980) *In vivo* tumor response to single and multiple exposures of adriamycin. *Eur. J. Cancer*, **16**, 1433.

SNEDECOR, G. W. & COCHRAN, W. G. (1973) *Statistical Methods*. Iowa: Iowa State University Press. p. 59.

SRIDHAR, R., KOCH, C. & SUTHERLAND, R. (1976) Cytotoxicity of two nitroimidazole radiosensitizers in an *in vitro* tumor model. *Int. J. Radiat. Oncol. Biol. Phys.*, **1**, 1149.

STEEL, G. G. & PECKHAM, M. J. (1979) Exploitable mechanisms in combined radiotherapy-chemotherapy: the concept of additivity. *Int. J. Radiat. Oncol. Biol. Phys.*, **5**, 85.

SUTHERLAND, R. M. (1974) Selective chemotherapy of noncycling cells in an *in vitro* tumor model. *Cancer Res.*, **34**, 3501.

SUTHERLAND, R. M., EDDY, H. A., BAREHAM, B., REICH, K. & VANANTWERP, D. (1979) Resistance to adriamycin in multicellular spheroids. *Int. J. Radiat. Oncol. Biol. Phys.*, **5**, 1225.

SUTHERLAND, R. M., BAREHAM, B. J. & REICH, K. A. (1980) Cytotoxicity of hypoxic cell sensitizers in multicell spheroids. *Cancer Clin. Trials*, **3**, 73.

TANNOCK, I. F. (1970) Population kinetics of carcinoma cells, capillary endothelial cells, and fibroblasts in a transplanted mouse mammary tumour. *Cancer Res.*, **30**, 2470.

THOMSON, J. E. & RAUTH, A. M. (1974) An *in vitro* assay to measure the viability of KHT tumor cells not previously exposed to culture conditions. *Radiat. Res.*, **58**, 262.

TWENTYMAN, P. R. (1977) Artefact introduced into clonogenic assays of bleomycin cytotoxicity. *Br. J Cancer*, **36**, 642.

TWENTYMAN, P. R., BROWN, J. M., GRAY, J. W., FRANKO, A. J., SCOLES, M. A. & KALLMAN, R. F. (1980) A new mouse tumor model system (RIF-1) for comparison end-point studies. *J. Natl Cancer Inst.*, **64**, 595.

WASSERMAN, T. H., PHILLIPS, T. L., JOHNSON, R. J. & 6 others (1979) Initial United States Clinical and pharmacologic evaluation of misonidazole (Ro-07-0582), a hypoxic cell radiosensitizer. *Int. J. Radiat. Oncol. Biol. Phys.*, **5**, 775.